# Primary tonsillar tuberculosis in a pediatric patient: Case report and literature review

**DOI:** 10.1097/MD.0000000000027616

**Published:** 2021-11-05

**Authors:** Stefana Maria Moisa, Ingrith Miron, Anca Adam-Raileanu, Vasile Valeriu Lupu, Ancuta Lupu, Elena Tarca

**Affiliations:** Department of Mother and Child Medicine – Pediatrics, “Grigore T. Popa” University of Medicine and Pharmacy, Iasi, Romania.

**Keywords:** a teenager, pediatrics, tonsillitis, tuberculosis

## Abstract

**Rationale:**

Tuberculosis is an entity that usually affects the lungs, although extrapulmonary sites can also be involved. Tonsils are rarely affected, especially in the absence of pulmonary disease, primary tonsillar tuberculosis being a diagnostic challenge for the clinician.

**Patient concerns:**

We present the case of a 14-year-old female teenager, presented to our Pediatric Service with a 14-day history of dysphagia, odynophagia and left reflex otalgia associated with a 5 kg weight loss. Clinical examination revealed mild pharyngeal erythema, marked enlargement of the left tonsil infiltrating the lateral pharyngeal wall and the uvula and painful, mobile, nonadherent to deep bilateral latero-cervical adenopathy.

**Diagnosis:**

Positive interferon-gamma release assay (QuantiFERON-TB gold). Mantoux test reading was 16 mm.

**Interventions:**

During hospitalization, the patient received Clindamycin and Gentamicin for 3 days i.v., with discrete relief of symptoms and inflammatory markers. On the 4th day of hospitalization, treatment with Imipenem/Cilastin is started for 7 days in micro-perfusion, with tonsil hypertrophy decrease in size and favorable clinical evolution.

**Outcome:**

Tonsil hypertrophy decreased in size and patient had a favorable clinical evolution. At discharge, the patient was given a 6-month course of anti-tuberculous drug.

**Lessons:**

The particularity of this case is represented by the rarity of primary tuberculosis of tonsils in children, with unilateral involvement, displaying at the same time a common issue encountered in the current practice: the limitations and the difficult course of setting the diagnosis due to the involvement of relatives in the medical act.

## 1 Introduction

Tuberculosis (TB) is an infectious malady frequently caused by Mycobacterium tuberculosis bacteria. Although it usually affects the lungs (90%), other organs can be involved, causing extrapulmonary tuberculosis.^[1]^ The most common extrapulmonary site of tuberculosis is represented by the lymph nodes, the nasopharynx and oropharynx, having a less than 5% incidence.^[2]^ Tonsils seem to acquire even in a smaller percentage of the disease due to the thickness of the protective epithelial covering of the oropharyngeal mucosa, to the oral cavity's saprophytes action or because of the antiseptic effect and cleansing action of the saliva.^[3]^ The right diagnosis and the accurate treatment are vital for peoples’ health, not ignoring the devastating social and economic impact, including poverty, stigma, and discrimination.

The most recent reports of the World Health Organization (WHO) show that 10 million people fell ill with TB in 2019, tuberculosis killing 5000 people every day. TB treatment saved about 58 million lives globally between 2000 and 2018, but important diagnostic and treatment gaps persist. Vaccination, pasteurization of milk to anti-TB treatments plays their role, but somehow the poor nutrition, low personal hygiene, immunodeficiency diseases and the increasing rates of multiple drug-resistant tuberculosis (MDR-TB) and extensively drug-resistant tuberculosis (XDR-TB) seem to frustrate the efforts of the healthcare providers.^[4]^ Pediatric tuberculosis in particular can be troublesome to discover and treat because often in everyday practice this diagnostic is overlooked. Another ugly truth of our days is that due to the current epidemiological context of “Sars-Cov-2 infection” the access to medical care tends to be more difficult and Mycobacterium tuberculosis tends to be forgotten, fact that inevitable leads to a delay in diagnosis and treatment.

In this study, we report a primary form of tonsillar tuberculosis, a rare medical condition, in a 14-year-old female accompanied by the medical literature.

## 2 Case report

We present the case of a 14-year-old female teenager, presented to our Pediatric Service with a 14-day history of dysphagia, odynophagia and left reflex otalgia associated with a 5 kg weight loss. During this time, the patient was treated with antibiotics (Amoxicillin/Clavulanate, Cefixime) and anti-inflammatory drugs, with no clinical improvement. There was no relevant personal or familial background of tuberculosis or other infectious. Immunized to date according to the national immunization scheme (BCG included).

On clinical examination, there was tender bilateral latero-cervical adenopathy (∼25/20 mm-right side, ∼30/25mm- left side), painful, mobile and nonadherent to the deep planes; mild posterior pharyngeal wall erythema, marked enlargement of the left tonsil infiltrating the lateral pharyngeal wall and the uvula, moderate trismus. No other abnormal findings were found on the detailed examination.

Initial laboratory tests showed normal complete blood count (CBC), a mildly elevated erythrocyte sedimentation rate (ESR) at 57 mm/1 h and serum C-reactive protein (CRP): 7.79 mg/L, normal liver, and renal function, quantitative immunoglobulins, and urinalysis. Negative pharyngeal swab.

Immunology evaluation identified high levels of Epstein–Barr antibodies to viral capsid antigen (VCA) IgG-66.58 S/CO, and Epstein–Barr nuclear antigen (EBNA) IgG-24.13 S/CO, elevated Cytomegalovirus IgM-1.4 and IgG antibodies >250 AU/mL.

To dispel the suspicion of either malignancy, either a rare infectious disease (Tuberculosis, Actinomycosis) a CT scan of the head and neck was needed, followed by a local biopsy—not possible due to the refusal of the patient's legal relatives. Biopsy was postponed. Instead, a soft tissue and abdominal ultrasound was performed: bilateral latero-cervical inflammatory lymph nodes, mesenteric adenitis, right inguinal inflammatory lymph node.

Positive interferon-gamma release assay (QuantiFERON-TB gold). Mantoux test reading was 16 mm. There were no acid-fast bacilli (AFB) detected from his morning gastric lavage fluid analysis, negative GeneXpert. Normal Chest X-ray excluded pulmonary involvement.

Craniocerebral and cervical magnetic resonance imaging (MRI): chronic bilateral maxillary sinusitis, bilateral ethmoiditis; inflammatory aspect at the level of bilateral palatine tonsils and adenoid vegetation; multiple bilateral upper and middle jugular lymphadenopathy.

During hospitalization, the patient received Clindamycin and Gentamicin for 3 days i.v., with discrete relief of symptoms and inflammatory markers. On the 4th day of hospitalization, treatment with Imipenem/Cilastin is started for 7 days in microperfusion, with tonsil hypertrophy decrease in size and favorable clinical evolution.

The case was diagnosed as a form of primary tonsillar tuberculosis. At discharge, the patient was given a 6-month course of anti-tuberculous drug.

## 3 Literature review methodology

A literature search was conducted using PubMed and Scopus to search case reports, clinical trials and case series published from January 1960 through September 2020 for the following keywords (tuberculosis or Mycobacterium tuberculosis) and tonsil. Scientific articles were selected and reviewed to assess the epidemiology, presentation, diagnosis and treatment of tonsillar tuberculosis. Studies reporting the simultaneous presence of tonsillar tuberculosis and tuberculosis of another site or oral malignancy in the same lesion site were also included. We excluded reports available only as abstracts or published in other language than English or Romanian.

The literature search identified 177 studies, of which 17 were not in English or Romanian, 45 concerned other sites of primary or secondary tuberculosis, and 52 were available only as abstracts. The remaining 63 studies consisted of 46 unique case reports and 17 case series, leaving 86 patients included in the analysis (Table 1).

**Table 1:** Main characteristics and results of the eligible studies.

Year	Author	Number of cases	Age (years)	Sex	Clinical symptoms	Tonsil affected	Latero-cervical adenopathy	AFB	Culture	Mantoux	Chest X-ray	Tonsilectomy	Anti-TB treatment	HIV	Tb concomitent involvment	Comorbidities
1964	Mohindra et al ^[20]^	4	NA	NA	frequent low fever	3 left tonsil 2 bilateral	Yes	pos	NA	1 poz	NA	Yes	NA	NA	Adenoids	NA
1966	Sanford et al ^[47]^	1	30	f	hoarseness difficulty in swallowing sore throat	bilateral	Yes	pos	pos	NA	pos	FNAC	Yes	neg	Lungs	No
1972	Cowan et al ^[44]^	1	48	m	persistent sore throat right otalgia dysphagia weight loss occasional night sweats	bilateral	Yes	pos	pos	poz	pos	FNAC	Yes	neg	Lungs	No
1979	Raman et al ^[32]^	1	41	m	left side pain difficulty swalowing	left	No	pos	neg	NA	pos	FNAC	Yes	NA	Lungs	Left tonsilar carcinoma
1987	Plesiss et al ^[9]^	1	6	f	sore throat fever anorexia left otalgia	bilateral	Yes	pos	neg	non reactive	pos	FNAC	Yes	neg	Lungs, larynx, mesenteric TB	No
1988	Park et al ^[55]^	1	20	f	sore throat hoarsness	left	No	pos	neg	NA	pos	FNAC	Yes	neg	Lungs, larynx, kidney	No
1989	Pedrol et al ^[36]^	1	22	m	progressive odynophagia	bilateral	No	pos	NA	pos	pos	Yes	Yes	neg	Lungs, pharynx	No
1994	Adiego et al ^[54]^	1	58	m	odynophagia dysphagia hyporexia weight loss	left	Yes	pos	pos	pos	pos	FNAC	Yes	neg	Lungs, Brain	No
1995	Selimoglu et al ^[18]^	1	67	m	sore throat	left	No	neg	NA	pos	neg	Yes	Yes	neg	–	No
2000	Sutbeyaz et al ^[56]^	1	48	m	Hoarseness difficulty in swallowing sore throat	left	No	pos	pos	pos	pos	FNAC	Yes	neg	Lungs, larynx	No
2000	Naik et al ^[29]^	1	18	f	recurrent throat pain fever	bilateral	No	neg	NA	neg	neg	Yes	Yes	neg	–	No
2000	Kardon et al ^[52]^	3	29, 34, 41	m	sore throat dysphagia nasal obstruction	bilateral	NA	pos	neg	neg	1 pos/2 neg	Yes	Yes	neg	Lungs	No
2000	Fortún et al ^[28]^	1	9	m	NA	bilateral	NA	pos	neg	NA	neg	Yes	Yes	NA	–	No
2001	Al-Serhani et al ^[63]^	5	NA	NA	sore throat throat discomfort	bilateral	2 Yes /3 No	NA	NA	NA	1 pos/4 neg	NA	NA	neg	1 lungs	No
2001	Awad et al ^[58]^	5	16-81	NA	sore throat dysphagia fever odynophagia	2 left tonsil/ 3 bilateral	3 Yes /2 No	pos	neg	2 pos	neg	FNAC	Yes	neg	–	No
2002	Yamamoto et al ^[57]^	1	23	f	sore throat hoarseness	bilateral	No	pos	neg	neg	pos	Yes	Yes	neg	Lungs, larynx	No
2002	Srirompotong et al ^[16]^	6	20-72	3m /3f	sore throat	NA	NA	5 pos/ 1 neg	NA	NA	4 pos/2 neg	Yes	Yes	neg	4 lungs	No
2003	Jana et al ^[42]^	1	20	m	persistent sore throat dysphagia cough dry	bilateral	Yes	pos	pos	NA	pos	FNAC	Yes	neg	Lungs	No
2003	Srirompotong et al ^[66]^	6	NA	NA	NA	NA	NA	NA	NA	NA	NA	NA	NA	NA	1 nasopharynx	No
2005	Efde et al ^[61]^	1	61	m	sore throat cough fever loss of appetite weight loss	left	No	pos	neg	pos	pos	Yes	Yes	neg	Lungs	Rheumatoid arthritis
2005	Nakayama et al ^[48]^	1	30	f	NA	left	Yes	pos	pos	pos	neg	Yes	Yes	neg	–	No
2006	Krishnappa et al ^[43]^	1	32	m	sore throat otalgia cough	left	Yes	pos	neg	neg	pos	FNAC	Yes	neg	Lungs	No
2006	Duijnstee et al ^[59]^	1	35	m	sore throat difficulty in swallowing weight loss	left	Yes	pos	pos	NA	neg	Yes	Yes	poz	–	HIV positive
2006	Ricciardiello et al ^[19]^	2	NA	NA	deteriorating dysphonia pharyngodynia	NA	NA	NA	NA	NA	pos	NA	Yes	neg	Lungs	No
2007	Maciaszczyk et al ^[53]^	1	45	f	sore throat anorexia fever cough	bilateral	Yes	pos	pos	NA	pos	Yes	Yes	neg	Lungs	No
2007	Al-Sebeih et al ^[33]^	1	65	m	NA	right	NA	pos	NA	pos	neg	Yes	NA	NA	Adenoids	NA
2008	Chakravarti et al ^[11]^	1	22	m	sore throat odynophagia occasional fever malaise	bilateral	No	pos	pos	NA	neg	FNAC	Yes	neg	–	No
2008	Belizna et al ^[46]^	1	40	m	sore throat dysphagia	bilateral	Yes	pos	pos	NA	pos	Yes	Yes	NA	Lungs	No
2008	Kant et al ^[31]^	1	55	m	sore throat difficulty in swallowing solid food	left	No	neg	pos	pos	neg	FNAC	Yes	neg	–	No
2010	Ghatak et al ^[41]^	1	7	f	odynophagia cough loss of appetite	left	Yes	neg	NA	neg	neg	FNAC	Yes	neg	–	No
2010	Barouta et al ^[38]^	1	67	m	NA	right	NA	neg	pos	poz	neg	FNAC	Yes	neg	–	rheumatoid arthritis
2010	Cherkaoui et al ^[30]^	5	15-42	3m /2f	dysphagia fever weight loss	bilateral	Yes 2	poz	NA	poz 3	poz 2	3 FNAC	Yes	neg	2 lungs	No
2011	McAllister et al ^[17]^	1	16	m	dysphagia	bilateral	Yes	pos	NA	NA	NA	Yes	NA	NA	–	NA
2012	Sood et al ^[13]^	1	58	f	stifness of mouth pain in throat bleeding from oral cavity	right	No	pos	neg	NA	neg	Yes	Yes	NA	–	No
2012	Balasubramanian et al ^[39]^	1	53	m	sore throat odynophagia loss of weight and appetite otalgia cough	right	Yes	neg	pos	NA	pos	FNAC	Yes	neg	Lungs	No
2012	Prasad et al ^[23]^	1	10	m	Fever difficulty in swallowing	bilateral	Yes	pos	pos	pos	neg	Yes	Yes	neg	–	No
2012	Bagga et al ^[63]^	1	31	m	severe burning non radiating pain cough loss of appetite	bilateral	Yes	neg	pos	NA	pos	FNAC	Yes	neg	Lungs	tongue cancer
2013	Lukšić et al ^[12]^	1	23	m	fever sore throat	bilateral	Yes	pos	neg	neg	neg	Yes	Yes	neg	–	No
2014	Barman et al ^[25]^	1	48	m	sore throat fever malaise	right	Yes	pos	NA	NA	neg	FNAC	Yes	neg	Cervical lymphadenitis	No
2014	Ceylan et al ^[65]^	1	56	m	sore throat dysphagia fatigue night sweats loss of weight	left	Yes	pos	pos	NA	pos	FNAC	Yes	neg	Larynx, lungs	No
2014	Ozbay et al ^[69]^	1	NA	NA	NA	bilateral	NA	pos	NA	NA	NA	Yes	NA	NA	–	NA
2014	Bruzgielewicz et al ^[62]^	7	49-71	NA	sore throat fever malaise weight loss	NA	Yes	pos	NA	3 pos	3 pos	3 FNAC/4 tonsilectomies	NA	NA	3 lungs	1 planoepithelial carcinoma
2015	Das et al ^[3]^	1	76	m	sore throat reccurent tossilitis progressive dysphagia	bilateral	Yes	pos	neg	NA	neg	FNAC and tonsilectomy	Yes	neg	–	No
2015	Kamath et al ^[34]^	1	20	f	sore throat fever progressive dysphagia	bilateral	Yes	pos	neg	NA	neg	FNAC and tonsilectomy	Yes	neg	Nasopharynx(adenoids)	No
2016	Kiliç et al ^[40]^	1	26	m	fever pain throat difficulty swalling weight loss	bilateral	No	pos	pos	NA	pos	Yes	Yes	neg	Lungs	No
2016	Amit et al ^[15]^	1	17	f	fever sore throat	bilateral	No	NA	neg	NA	neg	Yes	Yes	neg	–	No
2016	Amaya-Tapia et al ^[37]^	1	46	m	dysphagia weight loss	bilateral	Yes	pos	neg	pos	neg	FNAC	Yes	neg	Mesenteric lymphadenitis	No
2017	Ferreira et al ^[60]^	1	32	f	odynophagia fever	left	Yes	pos	neg	NA	pos	FNAC	Yes	neg	Lungs	Behçet's syndrome
2017	Sellami et al ^[27]^	1	36	f	sore throat progressive dysphagia	right	Yes	pos	neg	pos	neg	FNAC	Yes	neg	–	No
2017	Sriram et al ^[35]^	1	NA	NA	fever swallowing of the neck	NA	Yes	NA	NA	NA	NA	FNAC	NA	NA	Adenoids	No
2018	Subramani et al ^[7]^	1	22	f	sore throat	left	Yes	pos	NA	pos	NA	FNAC	Yes	neg	–	No
2018	Eroğlu et al ^[24]^	1	51	f	throat pain dysphagia loss of appetite	left	Yes	pos	pos	NA	neg	Yes	Yes	neg	–	No
2018	Sasikumar et al ^[22]^	1	22	f	post partum odynophagia fever loss of weight	left	Yes	pos	neg	pos	neg	Yes	Yes	neg	–	No
2019	Balica et al ^[21]^	1	44	m	intense odynophagia referred otalgia anterior uveitis	left	No	pos	neg	neg	neg	FNAC	Yes	neg	–	Conjunctival-palpebral Kaposi's sarcoma glaucoma anterior ischemic optic neuropathy
2019	Motahari et al ^[5]^	1	18	f	sore throat fever loss of weight	bilateral	Yes	pos	neg	neg	neg	Yes	Yes	neg	–	No
2019	Lim et al ^[2]^	1	6	m	recurrent sore throat associated with snoring	bilateral	Yes	pos	neg	poz	neg	Yes	Yes	neg	Adenoids	No
2020	Hassan et al ^[26]^	1	21	m	sore throat difficulty swaling odynohagia	bilateral	Yes	pos	NA	NA	neg	Yes	Yes	neg	–	No
2020	Dhanasekar T et al ^[1]^	1	10	m	throat pain snoring change in voice	bilateral	No	pos	neg+gene xpert	NA	neg	Yes	Yes	neg	–	No
2020	Tisekar et al ^[6]^	1	55	m	sore throat difficulty in swallowing	left	Yes	pos	NA	NA	neg	Yes	Yes	neg	–	No
2020	Naserrudin et al ^[10]^	1	26	m	recurrent sore throat episodes	bilateral	NA	pos	NA	NA	neg	Yes	Yes	neg	–	No
2020	Kayabasi et al ^[14]^	1	NA	f	NA	unilateral	NA	pos	NA	NA	neg	Yes	NA	NA	–	No

AFB = acid fast bacilli, f = female, FNAC = fine needle aspiration cytology, m = male, NA = not available, neg = negative, pos = positive.

## 4 Discussion

Tonsillar tuberculosis is a rare medical condition, both in children and adults. In addition to the general immune-suppressing conditions known to influence the incidence of TB in its pulmonary form, poor oral hygiene, periodontitis, dental extraction and leukoplakia seem to be predisposing factors for tuberculous tonsillitis.^[5]^ Its primary form is presented only as a tonsillar involvement without pulmonary touch.^[6]^ Medical history and clinical examination may raise the suspicion of tuberculosis. TST and IGRA provide evidence for infection but for confirmation, histological examination, Ziehl Neelsen staining, and mycobacterial culture must be done. Treatment of primary tonsillar tuberculosis consists of antituberculous therapy, 6 months of standard anti-tuberculous regimen.^[4,6]^ Although due to the atypical clinical appearance there is a delay in treatment initiation, there is a general good outcome when treated.^[7]^ Unlike its pulmonary form, tonsillar tuberculosis is a multidrug-resistant TB strain free, no case being reported in the literature.^[8]^

Related to our literature search, of the 63 included studies, 66.67% were conducted in Asia, 22.22% in Europe, 5.56% USA, 3.7% in Africa, and 1.85% in Australia, leaving Asia in a front position in TB incidence and total number of cases, just as WHO reports described. Asia had 44% of the new cases diagnosed in 2019, with the highest incidence of 4.34. The age varied from 3 to 80 years with a mean age of 34.9. Most tonsillar tuberculosis cases were identified among 20 to 50 years old adults: 62.74%, with a preponderance of male cases—67.25%, while women represented 37.25%. The male sex seemed predominant as well in the pediatric population (15.6% children < 18 years), were was identified only 1 case of secondary tonsillar tuberculosis.^[9]^ Findings are similar to WHO's distribution of cases reported in 2019: 56% men ≥15 years old, 32% women ≥15 years old, 12% children <15 years old.^[4]^

The commonest presentation of TB tonsil was sore throat (59%) just as Nadia Syafeera Naserrudin et al^[10]^ also observed. 15.11% of tonsils infected with TB appear to have ulceration, masses and white patches. In 46% of cases, both tonsils were involved, although a similar percentage of 41.2% of left tonsil lesions was found. Latero-cervical adenopathies were identified in 61.5% of patients. Isolated palatine tonsil lesions are acquired by inhalation and many times are related to pure oral hygiene or are seen in patients with a low immune status.^[11]^ Our review of literature identified only 7% of immunocompromised patients, most of the tonsillar tuberculosis cases being free of comorbidities, just as the presented patient.

All patients had diagnostic based on histopathological examination (54.4% tonsil-lectomy, 45.6% fine needle aspiration biopsy) with 95.58% positive results. Only 40 of them had their sputum examined with 42.5% positive direct smear microscopy. A tuberculosis skin test (TST) was conducted in 33 patients with 60.60% positive results. Forty-one percent of patients had pulmonary form of tuberculosis was confirmed by a positive chest X-ray, only 1 patient confirmed by CT-scan after initial negative X-ray assessment.

Primary tuberculosis of tonsils in the absence of pulmonary involvement is a rare entity with an incidence of <5%, although our literature review identified more cases of primary tonsillar tuberculosis than secondary forms (59% vs 41%): primary tonsillar tuberculosis was found in 59% of patients, 39.4% of them presenting tonsils as unique tuberculosis site^[3,10,12–31]^ while the rest of 9.6% of them had simultaneous tuberculosis and epithelial squamous cell carcinoma of the tonsil—1 case,^[32]^ 4 cases of tuberculosis of tonsil and adenoids,^[2,30–35]^ 3 cases of tonsillar and pharyngeal tuberculosis,^[9,20,36]^ 1 case of tonsillar and cervical lymphadenitis related to tuberculosis,^[25]^ 2 cases of tuberculosis involving the tonsils, cervical and mesenteric lymphadenitis,^[9,37]^ and 1 case of tonsillar tuberculosis developed under anti-TNF alfa inhibitors and Methotrexate treatment.^[38]^

The percentage of patients with concomitant pulmonary involvement was 41,^[10,16,39–53]^ among them identifying 1 case of simultaneous pulmonary, tonsillar and cerebral tuberculosis,^[54]^ 1 case of simultaneous pulmonary, tonsillar and renal involvement,^[55]^ 3 cases of pulmonary, tonsillar and larynx tuberculosis,^[56–58]^ and 5 immunocompromised patients: 1 case of HIV infection associated with pulmonary and tonsillar tuberculosis,^[59]^ 2 patients developing pulmonary and tonsillar tuberculosis under anti TNF alpha inhibitors treatment,^[60,61]^ and 2 cases of epithelial squamous cell carcinoma of the tonsil^[62]^ or tongue^[63]^ coexisting with pulmonary and tonsillar tuberculosis.

All patients received anti TB treatment, just as our presented case. Almost a quarter of them (26.7%) received anterior antibiotic treatment without any clinical improvement, exactly as our 14 old patient who took both betalactam antibiotics and cephalosporins at home, and lincosamide associated with an aminoglycoside during hospital admission, responding only to imipenem/cilastin, a 5th category of antituberculosis drug.^[64–69]^

Regarding our case report, we must admit that we had some limitations and the patient's medical management was not the one initially desired. We had a strong clinical suspicion of either malignancy, either infectious disease but despite explaining the patient's relatives the risks that inadequate treatment could expose the patient to, we faced their refusal for a CT scan, forward making a biopsy impossible to obtain. We had no other option than to wait and see the evolution under antibiotic treatment. After a MRI scan was performed, its result with the local clinical remission no longer recommended a local biopsy, leaving us no other way than establishing a diagnosis based only on a positive TST and QuantiFERON-TB gold result.

While both the patient's and doctor's autonomy are a dynamic interface influencing decision making, we faced the ethical dilemma of respecting patient's legal tutors right to refuse investigation knowing the risks that the patient could be exposed to. Unfortunately, an increasing number of patients tend to make an abuse of their legal rights because of present media propaganda, finally leading only to longer admission duration, higher costs and of course, more severe medical cases.^[70,71]^

The purpose of the present paper, a precise analysis of previous presented cases is to come as a support for the medical practitioners’ global efforts of reducing the TB medical, social and economic burden.

## 5 Conclusion

Primary tonsillar tuberculosis is a rare condition, especially in the pediatric age but must be considered when facing a persistent tonsillitis in countries with a high prevalence of tuberculosis. Histopathological findings and bacteriological confirmation are ideal for establishing a positive diagnosis and immediate initiation of adequate treatment. The particularity of this case is represented by the rarity of primary tuberculosis of tonsils in children, with unilateral involvement, displaying at the same time a common issue encountered in the current practice: the limitations and the difficult course of setting the diagnosis due to the involvement of relatives in the medical act.

## Acknowledgments

All authors have equally contributed in preparing this manuscript.

We like to thank the Radiology and Laboratory Department for their help in managing this patient.

## Author contributions

**Conceptualization:** Stefana Maria Moisa, Ingrith Miron, Anca Adam Raileanu, Vasile Valeriu Lupu, Ancuta Lupu, Elena Tarca.

**Investigation:** Stefana Maria Moisa, Ingrith Miron, Anca Adam Raileanu, Vasile Valeriu Lupu, Ancuta Lupu, Elena Tarca.

**Methodology:** Stefana Maria Moisa, Ingrith Miron, Anca Adam Raileanu, Vasile Valeriu Lupu, Ancuta Lupu, Elena Tarca.

**Writing – original draft:** Stefana Maria Moisa, Ingrith Miron, Anca Adam Raileanu, Vasile Valeriu Lupu, Ancuta Lupu, Elena Tarca.

**Writing – review & editing:** Stefana Maria Moisa, Ingrith Miron, Anca Adam Raileanu, Vasile Valeriu Lupu, Ancuta Lupu, Elena Tarca.
